# Dimer/monomer status and *in vivo* function of salt‐bridge mutants of the plant UV‐B photoreceptor UVR8

**DOI:** 10.1111/tpj.13260

**Published:** 2016-09-09

**Authors:** Monika Heilmann, Christos N. Velanis, Catherine Cloix, Brian O. Smith, John M. Christie, Gareth I. Jenkins

**Affiliations:** ^1^ Institute of Molecular, Cell and Systems BiologyCollege of Medical, Veterinary and Life SciencesUniversity of GlasgowBower BuildingGlasgowG12 8QQUK; ^2^ Present address: BASF SECrop ProtectionSpeyerer Str. 267117LimburgerhofGermany; ^3^ Present address: Cancer Research UK Beatson InstituteGarscube Estate, Switchback Road, BearsdenGlasgowG61 1BDUK

**Keywords:** UV‐B, UVR8, photoreceptor, photomorphogenesis, *Arabidopsis thaliana*

## Abstract

UV RESISTANCE LOCUS8 (UVR8) is a photoreceptor for ultraviolet‐B (UV‐B) light that initiates photomorphogenic responses in plants. UV‐B photoreception causes rapid dissociation of dimeric UVR8 into monomers that interact with CONSTITUTIVELY PHOTOMORPHOGENIC1 (COP1) to initiate signal transduction. Experiments with purified UVR8 show that the dimer is maintained by salt‐bridge interactions between specific charged amino acids across the dimer interface. However, little is known about the importance of these charged amino acids in determining dimer/monomer status and UVR8 function in plants. Here we evaluate the use of different methods to examine dimer/monomer status of UVR8 and show that mutations of several salt‐bridge amino acids affect dimer/monomer status, interaction with COP1 and photoreceptor function of UVR8 *in vivo*. In particular, the salt‐bridges formed between arginine 286 and aspartates 96 and 107 are key to dimer formation. Mutation of arginine 286 to alanine impairs dimer formation, interaction with COP1 and function *in vivo*, whereas mutation to lysine gives a weakened dimer that is functional *in vivo*, indicating the importance of the positive charge of the arginine/lysine residue for dimer formation. Notably, a UVR8 mutant in which aspartates 96 and 107 are conservatively mutated to asparagine is strongly impaired in dimer formation but mediates UV‐B responses *in vivo* with a similar dose–response relationship to wild‐type. The UV‐B responsiveness of this mutant does not correlate with dimer formation and monomerisation, indicating that monomeric UVR8 has the potential for UV‐B photoreception, initiating signal transduction and responses in plants.

## Significance Statement

The plant photoreceptor UVR8 mediates photomorphogenic responses to UV‐B. When the homo‐dimeric UVR8 protein absorbs UV‐B, interactions between charged amino acids that maintain the dimer structure are neutralized, causing monomerisation and initiating signal transduction. Here we determine the importance of specific charged amino acids in promoting dimer formation and photoreceptor function, but show that particular mutant proteins strongly impaired in dimerisation are still capable of mediating responses to UV‐B *in vivo*, indicating that monomeric UVR8 can be active in photoreception.

## Introduction

Ultraviolet‐B (UV‐B) wavelengths (280–315 nm) in sunlight have a major impact on the biosphere. The high energy of UV‐B radiation has the potential to damage molecules such as DNA, impair cellular activities and cause cell death. However, plants have evolved effective mechanisms to protect themselves from damage by UV‐B, which enable them to survive constant exposure to sunlight. In particular, plants synthesize UV‐absorbing sunscreen compounds that are deposited in the outer tissues, and they employ efficient cellular systems for repairing damage by UV‐B (Frohnmeyer and Staiger, 2003; Ulm and Nagy, 2005; Jenkins, 2009). The exposure of plants to low doses of UV‐B stimulates transcriptional responses that underpin UV‐protection (Ulm *et al*., 2004; Brown *et al*., 2005; Favory *et al*., 2009). Furthermore, UV‐B acts as a regulatory stimulus for other responses in plants, including the suppression of extension growth (Favory *et al*., 2009; Hayes *et al*., 2014), entrainment of the circadian clock (Feher *et al*., 2011) and defence against insect herbivory (Ballaré *et al*., 2012).

Regulatory responses to UV‐B are mediated by the photoreceptor UV RESISTANCE LOCUS8 (UVR8; Brown *et al*., 2005; Favory *et al*., 2009; Rizzini *et al*., 2011; Tilbrook *et al*., 2013; Jenkins, 2014a). Arabidopsis *uvr8* mutant plants are defective in photomorphogenic responses to UV‐B and are highly susceptible to damage by UV‐B because they are unable to stimulate expression of genes concerned with UV‐protection (Kliebenstein *et al*., 2002; Brown *et al*., 2005; Favory *et al*., 2009). UVR8 is a 7‐bladed β‐propeller protein that is present as a homo‐dimer in plants (Rizzini *et al*., 2011; Christie *et al*., 2012; O'Hara and Jenkins, 2012; Wu *et al*., 2012; Jenkins, 2014b). Unlike other photoreceptors, UVR8 does not use a prosthetic chromophore for light sensing, instead specific tryptophan amino acids of UVR8 act as chromophores for UV‐B detection (Rizzini *et al*., 2011; Christie *et al*., 2012; O'Hara and Jenkins, 2012; Wu *et al*., 2012; Zeng *et al*., 2015). Photoreception induces rapid dissociation of the dimer into monomers (Rizzini *et al*., 2011; Christie *et al*., 2012; Wu *et al*., 2012), which then interact with the CONSTITUTIVELY PHOTOMORPHOGENIC1 (COP1) protein to initiate signaling and hence regulate transcription of target genes involved in UVR8‐mediated responses (Favory *et al*., 2009; Rizzini *et al*., 2011; Cloix *et al*., 2012). One of the genes most rapidly induced by UV‐B following UVR8 photoreception encodes the transcription factor ELONGATED HYPOCOTYL5 (HY5). This transcription factor, sometimes acting with the related HY5 HOMOLOG (HYH) is a key effector of transcriptional responses regulated by UVR8 (Brown *et al*., 2005; Oravecz *et al*., 2006; Brown and Jenkins, 2008; Favory *et al*., 2009; Huang *et al*., 2013). In addition, UVR8 photoreception stimulates expression of genes encoding the REPRESSOR OF UV PHOTOMORPHOGENESIS1 (RUP1) and RUP2 proteins, which negatively regulate UVR8 responses (Grüber *et al*., 2010).

The crystal structure of UVR8 shows that dimer formation is dependent on salt‐bridge interactions between charged amino acids at the interface where monomers come into contact (Christie *et al*., 2012; Wu *et al*., 2012; Jenkins, 2014b). Hydrophobic interactions between the monomers are negligible. The dimerisation surface of each monomer is rich in basic amino acids, notably arginine, and acidic aspartate and glutamate residues. These amino acids are distributed such that patches of complementary electrostatic potential are aligned opposite each other across the dimer interface. The salt‐bridge interactions are sufficiently strong that the dimer is maintained even in the presence of SDS, as long as the protein sample is not boiled (Rizzini *et al*., 2011; Christie *et al*., 2012; Wu *et al*., 2012). However, monomerisation occurs when salt‐bridges are neutralized by low pH and high ionic strength (Christie *et al*., 2012; Wu *et al*., 2012). Moreover, studies with purified UVR8 expressed in *Escherichia coli* show that mutation of particular salt‐bridging amino acids prevents dimer formation (Christie *et al*., 2012; Wu *et al*., 2012).

Inevitably the molecular environment of UVR8 in cells will differ from that *in vitro*. It is therefore essential to establish whether findings with the purified protein extend to UVR8 in plants. Hence, the aim of the present study was to determine whether particular salt‐bridge amino acids are important in maintaining UVR8 structure in plants and to assess the consequences of altered dimer/monomer status on UVR8 function *in vivo*. We show that specific arginine and aspartate amino acids at the UVR8 dimerisation surface are required for dimer formation in transgenic Arabidopsis plants. However, although mutants in some of these amino acids are impaired in dimer formation both *in vitro* and *in vivo*, they are nevertheless able to functionally complement Arabidopsis *uvr8‐1* plants. This study highlights the methodological difficulty of establishing the dimer/monomer status of a UVR8 mutant protein in plants, and provides evidence that UVR8 can perceive UV‐B and initiate signaling even in its monomeric form.

## Results

### Dimer/monomer status of selected UVR8 salt‐bridge mutant proteins

**Figure 1:**
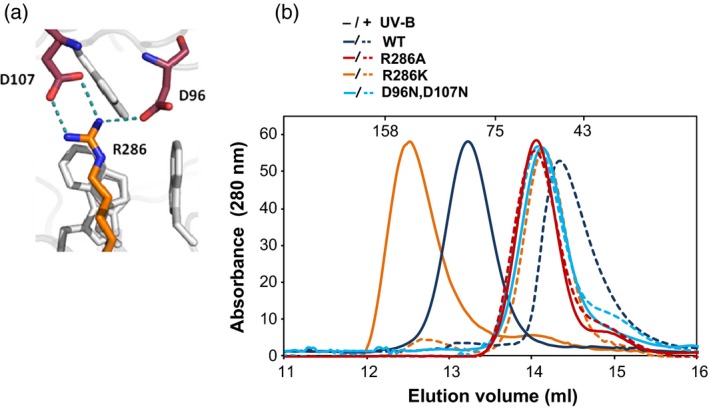
Dimer/monomer status of purified UVR8 salt‐bridge mutant proteins. (a) PyMol image showing inter‐monomer salt‐bridges formed between R286 and D96 and D107. (b) Size exclusion chromatography on a Superdex 200 column of purified wild‐type UVR8 and the UVR8^R286A^, UVR8^R286K^ and UVR8^D96N,D107N^ mutant proteins exposed (dashed line) or not (solid line) to 1.5 μmol m^−2^ sec^−1^ narrowband UV‐B for 1 h. Elution points of marker proteins (in kDa) are shown at the top.

We wished to study the effects of mutations of several charged amino acids at the dimer interface on UVR8 dimer/monomer status *in vivo*. The selection of amino acids and mutations was based on examination of the UVR8 crystal structure and on biochemical studies of purified UVR8 mutant proteins, which identified amino acids that are critical for dimer formation *in vitro* (Christie *et al*., 2012; Wu *et al*., 2012). A key residue is arginine R286, which is adjacent to the principal chromophore tryptophan, W285, and forms double and single H‐bonded salt‐bridges, respectively, with aspartates D107 and D96 (Figure 1a). Size exclusion chromatography (SEC) shows that purified wild‐type UVR8 is a dimer when not exposed to UV‐B and a monomer following UV‐B exposure, whereas mutation of either R286 to alanine (UVR8^R286A^) or D96 and D107 to asparagine (UVR8^D96N,D107N^) causes UVR8 to become constitutively monomeric *in vitro* (Figure 1b; Christie *et al*., 2012; Wu *et al*., 2012). In contrast, UVR8^R286K^, in which R286 is conservatively mutated to positively charged lysine, appears dimeric and monomerises in response to UV‐B (Figure 1b). The SEC elution volume of the UVR8^R286K^ dimer differs from that of wild‐type UVR8, most likely because of a change in the hydrodynamic radius (shape) of the protein; this difference is not evident when the salt concentration is increased (Figure S1). Similar to R286, R146 forms a double H‐bonded salt‐bridge, in this case with E182 (Figure S2a). However, in contrast to UVR8^R286A^, UVR8^R146A^ is a dimer *in vitro* that monomerises in response to UV‐B (Christie *et al*., 2012; Wu *et al*., 2012; Figure S2b). R234, which is adjacent to UV‐B chromophore W233, forms an intra‐molecular salt‐bridge with E182 (Figure S2a). UVR8^R234A^ adopts a conformation *in vitro* that is non‐responsive to UV‐B (Figure S2c), most likely because the mutation could disrupt the spatial arrangement of the chromophore tryptophans, impairing UV‐B photoreception. R338 is adjacent to the triad tryptophan W337 and forms a single hydrogen‐bonded salt‐bridge with D44 and a non hydrogen‐bonded ionic interaction with E43, as well as a water mediated hydrogen bond with its backbone carbonyl (Figure S3a). The UVR8^R338A^ mutant is reported to be constitutively monomeric *in vitro* (Wu *et al*., 2012). However, the dimer/monomer status of UVR8^R338A^ *in vitro* is dependent on the salt concentration; it is monomeric in 500 mm NaCl but appears to be in equilibrium between dimer and monomer in low salt concentrations (Figure S3b).

### Mutation of key UVR8 salt‐bridge amino acids impairs dimer formation in plants

**Figure 2:**
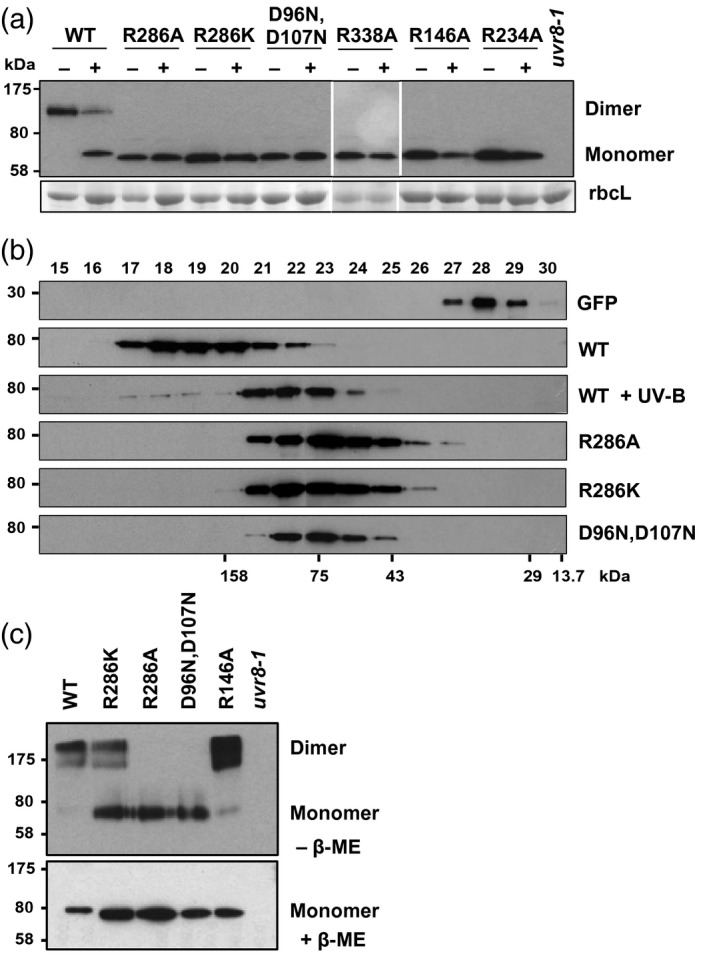
Dimer/monomer status of UVR8 salt‐bridge mutant proteins expressed in plants. (a) Western blot of whole cell extracts from *uvr8‐1* plants expressing either GFP‐UVR8 or GFP‐UVR8 salt‐bridge mutants exposed (+) or not (−) to 4 μmol m^−2^ sec^−1^ narrowband UV‐B for 30 min. SDS‐loading buffer was added and samples were run on a 7.5% SDS–PAGE gel without boiling. An immunoblot was probed with anti‐UVR8 antibody. Ponceau staining of Rubisco large subunit (rbcL) is shown as a loading control. The GFP‐UVR8 dimer and monomer are indicated. (b) Size exclusion chromatography profiles of immunoprecipitated wild‐type GFP‐UVR8 (WT) and salt‐bridge mutant fusions expressed in *Nicotiana benthamiana* plants. Vector with GFP alone was used as a control. For wild‐type GFP‐UVR8, extracts were illuminated (or not) with 4 μmol m^−2^ sec^−1^ narrowband UV‐B for 30 min. All other samples were not exposed to UV‐B. Eluates of immunoprecipitation assays with anti‐GFP beads were loaded onto a Superdex 200 column, and fractions 15–30 were used for standard SDS–PAGE and immunoblotting with an anti‐GFP antibody. (c) Western blot of whole cell extracts from *uvr8‐1* plants expressing either GFP‐UVR8 (WT) or GFP‐UVR8 salt‐bridge mutants not exposed to UV‐B incubated with the cross‐linking reagent dithiobis (succinimidylpropionate) (DSP) in the absence (upper panel) or presence (lower panel) of β‐mercaptoethanol (β‐ME). SDS‐loading buffer was added and samples were run on a 10% SDS–PAGE gel without boiling. An immunoblot was probed with anti‐UVR8 antibody. The UVR8 dimer and monomer are indicated.

The mutant UVR8 proteins described above were expressed as GFP fusions in the null *uvr8‐1* mutant. Several transgenic lines were obtained for each mutant and compared with a control GFP‐UVR8 fusion that was shown previously to functionally complement *uvr8‐1* (Brown *et al*., 2005; Kaiserli and Jenkins, 2007; Figure S4). The dimer/monomer status of the mutant UVR8 proteins was examined initially using SDS–PAGE with non‐boiled samples. This assay shows that wild‐type GFP‐UVR8 is a dimer that monomerises after UV‐B exposure, but in contrast each mutant protein appears constitutively monomeric (Figure 2a). However, this method very sensitively detects even slight weakening of the dimer and does not rigorously determine dimer/monomer status; for instance, purified UVR8 mutant proteins, such as UVR8^R146A^ and UVR8^R286K^, which appear dimeric in the absence of UV‐B when examined by SEC (Figures 1b and S2b), appear constitutively monomeric in the SDS–PAGE assay (Figure S5). We therefore used additional methods to examine dimer/monomer status of the mutant proteins.

We expressed each GFP‐tagged UVR8 mutant protein transiently in Nicotiana leaves, immunoprecipitated the protein from an extract and used SEC to determine its dimer/monomer status. In this assay, wild‐type GFP‐UVR8 protein is dimeric in the absence of UV‐B but is monomeric following exposure of the protein extracts to UV‐B (Figure 2b). In contrast, each of the mutant proteins is constitutively monomeric in this assay. However, the conditions used to obtain immunoprecipitated UVR8 could promote monomerisation of mutants with weak dimers and so the results may not reflect the dimer/monomer status of the mutant proteins *in planta*.

We therefore used cross‐linking with dithiobis(succinimidylpropionate) (DSP), to establish whether UVR8 mutant proteins are dimeric or monomeric in plants. This method has been used previously to show that wild‐type UVR8 forms a dimer that dissociates into monomers following UV‐B exposure (Rizzini *et al*., 2011). In the experiment shown in Figure 2(c), the cross‐linking agent was added to protein extracts of plants not exposed to UV‐B. Wild‐type GFP‐UVR8 is dimeric in this assay, as is GFP‐UVR8^R146A^; GFP‐UVR8^R286K^ appears as a dimer with some monomer present, consistent with SEC of the purified protein at elevated salt concentrations (Figure S1). However, for both GFP‐UVR8^R286A^ and GFP‐UVR8^D96N,D107N^, only monomeric protein is detectable.

### Some, but not all, salt‐bridge mutants interact constitutively with COP1

UV‐B photoreception stimulates monomerisation and interaction of UVR8 with COP1 to initiate signaling (Rizzini *et al*., 2011). COP1 interacts with a 27 amino acid region near the C‐terminus of UVR8 and also with the β‐propeller core of the protein (Cloix *et al*., 2012; Yin *et al*., 2015). It is proposed that the C‐terminus becomes accessible to COP1 following UV‐B exposure of UVR8 (Cloix *et al*., 2012). However, some tryptophan mutants of UVR8 bind COP1 in the absence of UV‐B (O'Hara and Jenkins, 2012; Heijde *et al*., 2013; Huang *et al*., 2013), suggesting that these mutations expose the C‐terminus. Because several of the salt‐bridge mutants have weakened dimers, we examined their interaction with COP1, which impacts on their potential ability to function.

**Figure 3:**
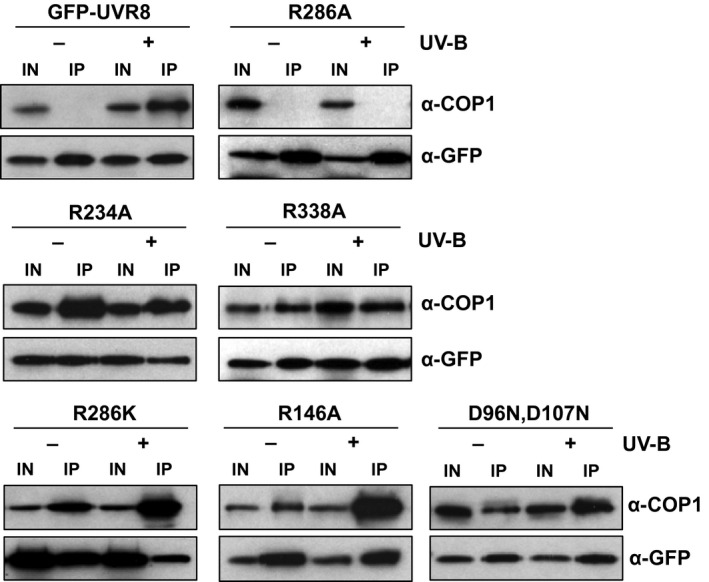
Interaction of UVR8 mutants with COP1 *in vivo*. Co‐immunoprecipitation of GFP‐UVR8 and COP1 in whole cell extracts obtained from *uvr8‐1* plants transformed with either GFP‐UVR8 or GFP‐UVR8 salt‐bridge mutants exposed (+) or not (−) to 3 μmol m^−2^ sec^−1^ narrowband UV‐B for 3 h. Co‐immunoprecipitation assays were performed under the same conditions. Input samples (15 μg, IN) and eluates (IP) were run on a SDS–PAGE gel, and an immunoblot was probed with anti‐COP1 and anti‐GFP antibodies.

We examined whether the salt‐bridge mutants were able to interact with COP1 using a co‐immunoprecipitation assay. As shown in Figure 3, GFP‐UVR8 interacted with COP1 in the presence but not the absence of UV‐B, as reported previously (Favory *et al*., 2009; Cloix *et al*., 2012). However, GFP‐UVR8^R286A^ did not interact with COP1 at all. In contrast, each of the other mutants tested interacted with COP1 in both the presence and absence of UV‐B. For GFP‐UVR8^R286K^ and GFP‐UVR8^R146A^, which have weakened dimers that monomerise in response to UV‐B, and monomeric GFP‐UVR8^D96N,D107N^, more COP1 was consistently co‐immunoprecipitated from UV‐B‐exposed plants.

### Some salt‐bridge mutants are functional in plants

To test whether the UVR8 mutants are functional in initiating photomorphogenic responses to UV‐B, we examined both the suppression of hypocotyl extension and the induction of gene expression, assaying specifically *HY5* and *CHS* transcript levels and CHS protein accumulation.

**Figure 4:**
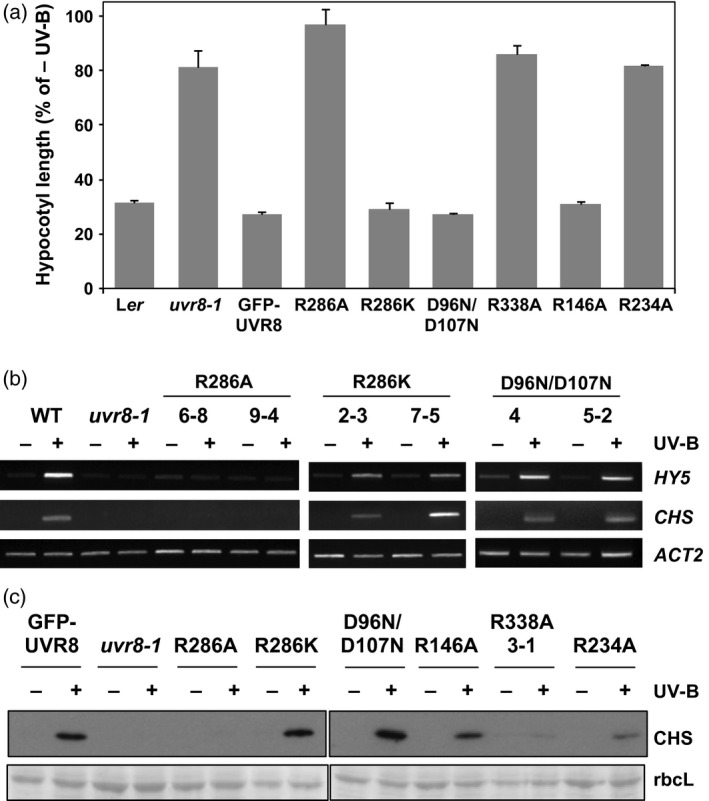
Functional complementation of *uvr8‐1* by UVR8 salt‐bridge mutant proteins. (a) Hypocotyl lengths (±SE,* n* = 10) for 4‐day‐old wild‐type L*er*,* uvr8‐1*, GFP‐UVR8 and the indicated transgenic lines of UVR8 salt‐bridge mutant seedlings grown in 1.5 μmol m^−2^ sec^−1^ white light (−UV‐B) supplemented with 1.5 μmol m^−2^ sec^−1^ narrowband UV‐B (+UV‐B). (b) RT‐PCR assays of *HY5*,*CHS* and control *ACTIN2* transcripts in L*er* (WT), *uvr8‐1*, and independent transgenic lines expressing either UVR8^R286A^, UVR8^R286K^ or UVR8^D96N,D107N^ grown under 20 μmol m^−2^ sec^−1^ white light (−) and exposed to 3 μmol m^−2^ sec^−1^ broadband UV‐B for 4 h (+). (c) Expression of CHS protein in GFP‐UVR8, *uvr8‐1* and the indicated transgenic lines of UVR8 salt‐bridge mutant plants grown and illuminated as in (b) for 7 days. Protein extracts were run on standard SDS–PAGE, and an immunoblot was probed with anti‐CHS antibody. Ponceau staining of Rubisco large subunit (rbcL) is shown as a loading control.

As shown in Figure 4(a), hypocotyl growth is suppressed by a low fluence rate of narrowband UV‐B in wild‐type plants and in the control GFP‐UVR8 transgenic line, but the response is impaired in *uvr8‐1* plants (Favory *et al*., 2009; Cloix *et al*., 2012). The GFP‐UVR8^R286A^, GFP‐UVR8^R338A^ and GFP‐UVR8^R234A^ mutants do not show growth suppression and are of similar length to *uvr8‐1* under UV‐B. In contrast, GFP‐UVR8^R286K^, GFP‐UVR8^R146A^ and GFP‐UVR8^D96N,D107^ show very similar hypocotyl growth suppression to wild‐type and GFP‐UVR8 plants, and are evidently functional in the UV‐B response. Very similar results were obtained for the induction of gene expression. *HY5* and *CHS* transcript accumulation are induced by UV‐B in two independent lines of the GFP‐UVR8^R286K^ and GFP‐UVR8^D96N,D107N^ mutants, but the GFP‐UVR8^R286A^ mutant fails to show induction (Figure 4b). Equivalent results were obtained for CHS protein accumulation in these mutants (Figure 4c). GFP‐UVR8^R146A^ also shows UV‐B induction of CHS, whereas GFP‐UVR8^R338A^ has little if any response, consistent with the hypocotyl suppression data. GFP‐UVR8^R234A^ shows a small response to UV‐B in CHS induction that is not apparent in hypocotyl growth suppression. The molecular and UV‐B response phenotypes of the UVR8 mutants are summarised in Table S1.

**Figure 5:**
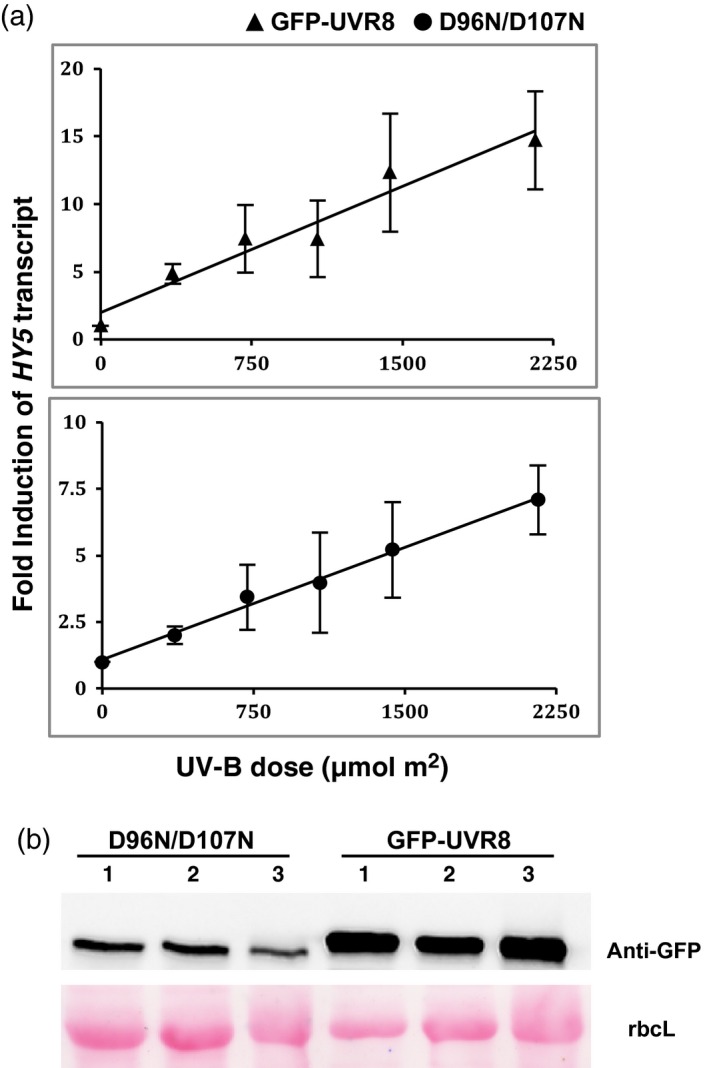
UV‐B dose–response of *HY5* transcript accumulation in UVR8^D96N,D107N^ mutant compared with GFP‐UVR8. (a) qRT‐PCR measurements of *HY5* transcripts in GFP‐UVR8 and GFP‐UVR8^D96N,D107N^ plants exposed to a range of doses of narrowband UV‐B. *HY5* transcripts were normalised to control *ACTIN2* transcript levels and fold‐induction is relative to the pre‐illumination dark transcript level. The data are the means (±SD) of three independent experiments. (b) Abundance of GFP‐UVR8^D96N,D107N^ and GFP‐UVR8 proteins in plants used for the three replicate experiments in (a). Protein extracts were subjected to SDS–PAGE and an immunoblot was probed with anti‐GFP antibody. Ponceau staining of Rubisco large subunit (rbcL) is shown as a loading control. Quantification of relative band intensities indicates that the mean level of GFP‐UVR8^D96N,D107N^ is approximately 25% that of GFP‐UVR8.

To further test whether the putatively monomeric GFP‐UVR8^D96N,D107N^ mutant is similarly responsive to UV‐B as wild‐type GFP‐UVR8, we examined the dose–response relationship for *HY5* transcript accumulation. We previously reported that this UV‐B response shows reciprocity between exposure duration and fluence rate (Brown *et al*., 2009), and used this information to select treatment conditions for the present study. The results (Figure 5) show that the GFP‐UVR8^D96N,D107N^ mutant and GFP‐UVR8 both respond to UV‐B with linear increases in *HY5* transcript levels over the same fluence range. The mutant has a lower fold‐induction of *HY5* transcripts, but this is likely because the plants used for these experiments expressed lower amounts of photoreceptor protein than the GFP‐UVR8 control (Figure 5b). It is known that the magnitude of response mediated by UVR8 is related to its level of expression (Favory *et al*., 2009).

**Figure 6:**
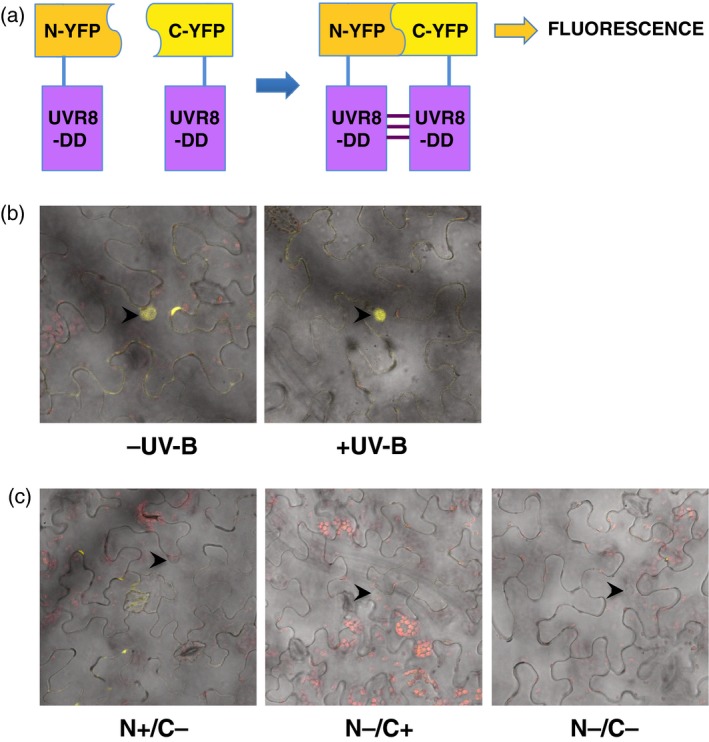
Bimolecular fluorescence complementation (BiFC) analysis of UVR8^D96N,D107N^ dimer/monomer status. (a) UVR8^D96N,D107N^ (UVR8‐DD) is expressed as a translational fusion with either the N‐ or C‐terminal region of YFP. Monomer interaction will lead to reconstitution of YFP and hence fluorescence. (b) Co‐transfection of plasmids expressing UVR8^D96N,D107N^/N‐YFP and UVR8^D96N,D107N^/C‐YFP in *Nicotiana benthamiana*. Confocal image of YFP fluorescence superimposed on bright‐field image of *N. benthamiana* epidermal cells. Plants were exposed to either UV‐B or no UV‐B following transfection. Arrows indicate nuclei. (c) Confocal images of control transfections: UVR8^D96N,D107N^/N‐YFP and empty vector/C‐YFP (N+/C−); empty vector/N‐YFP and UVR8^D96N,D107N^/C‐YFP (N−/C+); empty vector/N‐YFP and empty vector/C‐YFP (N−/C−). Arrows indicate nuclei.

Because the putatively monomeric GFP‐UVR8^D96N,D107N^ mutant mediated responses to UV‐B similarly to wild‐type GFP‐UVR8, we further examined whether the mutant fails to form dimers *in vivo*. We used a sensitive bimolecular fluorescence complementation (BiFC) assay (Walter *et al*., 2004) in which the protein is transiently expressed in Nicotiana leaves as a fusion with either the N‐ or C‐terminal region of YFP (Figure 6a). Dimer formation is expected to permit reconstitution of YFP and hence generate a fluorescence signal, whereas no signal should be seen if the mutant protein is constitutively monomeric. Expression of either the N‐ or C‐terminal fusion protein together with the complementary expression vector containing no fusion protein is used as a control. As shown in Figure 6(b), a fluorescence signal is detected for UVR8^D96N,D107N^ predominantly in the nucleus under both minus and plus UV‐B conditions, whereas no fluorescence is seen with the empty‐vector controls (Figure 6c). Similar results were obtained for UVR8^D96N,D107N^ in six independent experiments.

## Discussion

Mutations of UVR8 salt‐bridge amino acids affect dimer stability to varying extents. However, the method used to assess dimer/monomer status is critical. SDS–PAGE with non‐boiled samples is convenient for monitoring the dimer/monomer status of wild‐type UVR8 in response to different treatments (Rizzini *et al*., 2011; Christie *et al*., 2012; Heijde and Ulm, 2013; Heilmann and Jenkins, 2013; Huang *et al*., 2014), but it has limited value for characterising mutant proteins because it very sensitively detects any reduction in affinity in the dimer. Thus, all salt‐bridge mutants examined to date appear constitutively monomeric when analysed by this method, either as purified proteins (Christie *et al*., 2012; Wu *et al*., 2012; Figure S5) or in plant extracts (Figure 2a). In contrast, SEC with purified proteins shows that some salt‐bridge mutants (UVR8^R286K^; UVR8^R146A^) are dimeric (Figures 1b and S2b) and are converted to monomers upon UV‐B illumination. Nevertheless, caution is required with the SEC analysis because some salt‐bridge mutations affect the hydrodynamic radius of the protein, and hence the elution volume (e.g. UVR8^R286K^; Figure 1b), most likely by altering the position of the flexible C‐terminal region. Moreover, the conformation of some mutants (e.g. UVR8^R338A^; Figure S3b) is significantly affected by ionic strength. It would be valuable to develop a quantitative method to evaluate the relative strength of the UVR8 dimer in different mutants by measuring a dissociation constant, but this has not yet been done for purified protein and extending the method to plant extracts would be difficult.

To assess the dimer/monomer status of salt‐bridge mutants *in vivo*, we attempted to employ SEC with immunoprecipitated, transiently expressed proteins. Using this method, wild‐type UVR8 appears as a dimer that monomerises after UV‐B exposure, as expected. In contrast, the salt‐bridge mutants appear constitutively monomeric. While this observation could suggest that the molecular environment *in vivo* impairs dimerisation of salt‐bridge mutants, it is more likely that the high pH used to elute the immunoprecipitated proteins causes dissociation of the weakened mutant dimers. A more reliable method to assess dimer/monomer status *in vivo* is cross‐linking of proteins, as it does not employ a chemical treatment that will promote monomerisation. The results obtained using cross‐linking are consistent with the SEC data for purified mutant proteins. In the absence of UV‐B, GFP‐UVR8^R146A^ is predominantly a dimer, whereas GFP‐UVR8^R286K^ is a mixture of dimer and monomer. In contrast, GFP‐UVR8^R286A^ and GFP‐UVR8^D96N,D107N^ are monomeric with no detectable dimer, again in agreement with the *in vitro* data. Nonetheless, although the data obtained with cross‐linking concur with those obtained for purified proteins, the assay is performed with protein extracts rather than intact cells, and it is important to determine the dimer/monomer status of the protein where it functions in cells.

The BiFC analysis of UVR8^D96N,D107N^ (Figure 6) contradicts the cross‐linking data in that dimers are detected in the nucleus. A possible explanation is that the amount of dimer formed by the mutant protein is below the limit of detection of the cross‐linking assay; nuclear UVR8 is estimated to be approximately 10% of the total UVR8 in wild‐type cells (Kaiserli and Jenkins, 2007), and probably an even smaller fraction of the UVR8^D96N,D107N^ protein in the nucleus is in the dimeric form. On the other hand, BiFC is very sensitive and the YFP interaction is irreversible, so the fluorescence observed may represent trapping of short‐lived, weak interaction between monomers of the mutant proteins. Thus, although BiFC shows that the UVR8^D96N,D107N^ mutant is capable of being trapped in a YFP linked state *in vivo*, the method may over‐state the extent and stability of dimer formation. The other methods employed all indicate that UVR8^D96N,D107N^ is constitutively monomeric, within their limits of detection, and it is therefore likely that this mutant forms very little dimer *in vivo*, and the dimers present are likely to be unstable and short lived.

We therefore conclude that mutations that affect the ability of purified UVR8 to form a dimer have an equivalent effect *in vivo* despite evident differences in the cellular environment, not least in ionic composition and the presence of proteins that could potentially influence dimer/monomer status. Furthermore, the *in vivo* experiments highlight the importance of specific charged amino acids in maintaining the dimer structure. In particular, the R286‐D96/D107 salt‐bridges are crucial for maintaining the dimer both *in vivo* and *in vitro*. The UVR8^D96N,D107N^ and UVR8^R286A^ mutants are strongly impaired in dimer formation, whereas UVR8^R286K^ forms a weakened dimer, indicating that the positive charge of the arginine/lysine residue is critical for dimerisation.

Assays of hypocotyl growth suppression and gene expression show that some salt‐bridge mutants are functional in response to UV‐B exposure. The impaired responses of GFP‐UVR8^R234A^, GFP‐UVR8^R286A^ and GFP‐UVR8^R338A^ to UV‐B may be because the mutations impact on photoreception. R234, R286 and R338 are adjacent to the triad tryptophans involved in UV‐B sensing, and the introduction of the small non‐polar alanine in place of arginine is likely to disrupt the spatial relationships of the tryptophans as well as charge‐based interactions between the tryprophans and adjacent arginines that are probably important in photoreceptor function (Wu *et al*., 2012). In contrast, photoreception is not impaired in the GFP‐UVR8^R146A^, GFP‐UVR8^R286K^ and GFP‐UVR8^D96N,D107N^ mutants, and the hypocotyl growth suppression and gene expression responses to UV‐B are very similar to those of wild‐type UVR8. These mutations should have little impact on UV‐B photoreception by UVR8. R146 is not close to the tryptophan triad and UVR8^R286K^ is a conservative mutation. D96 and D107 are not adjacent to the chromophore tryptophans and are not part of the charge network that surrounds them (Christie *et al*., 2012; Wu *et al*., 2012), and because aspartate and asparagine are very similar in size the UVR8^D96N,D107N^ mutations will not cause spatial disruption within the monomer.

Interaction of UVR8 with COP1 is necessary to initiate signaling and transcriptional responses. The GFP‐UVR8^R286A^ mutant is unable to bind COP1 and is non‐functional, consistent with the findings of Huang *et al*. (2014). A possible explanation is that this mutation prevents UV‐B‐induced conformational changes that make the C‐terminus, and potentially other regions of the protein, accessible for binding to COP1. In contrast, the constitutive binding of COP1 to GFP‐UVR8^D96N,D107N^ is likely caused by exposure of residues involved in binding. Similarly, the binding of COP1 to the weakened dimers of GFP‐UVR8^R146A^ and GFP‐UVR8^R286K^ is presumably the result of physical exposure of the region(s) interacting with COP1 in the non‐illuminated photoreceptor as a result of the mutations. Although several of the mutants studied here bind COP1 constitutively, none initiate responses in the absence of UV‐B, indicating that interaction with COP1 is not sufficient to initiate signaling. Similarly, the constitutive binding of COP1 to GFP‐UVR8^R234A^ and GFP‐UVR8^R338A^ is not sufficient for function. These results are consistent with those reported previously for alanine mutants of triad tryptophans (O'Hara and Jenkins, 2012). In contrast, strong over‐expression of UVR8^W285A^ gives a *cop* mutant phenotype, likely because of sequestration of COP1, and the plants show constitutive activation of UV‐B signaling (Heijde *et al*., 2013). A similar phenotype is reported for UVR8^R338A^ (Huang *et al*., 2014), in contrast to the data in Figure 4. A possible explanation of these different findings is that the lower level of transgenic expression employed in this study and previously (Kaiserli and Jenkins, 2007; O'Hara and Jenkins, 2012) is too low to promote obvious constitutive activation.

The finding that the *in vivo* UV‐B responses of GFP‐UVR8^D96N,D107N^ are very similar to those of wild‐type GFP‐UVR8 is particularly interesting, because the mutant is evidently strongly impaired in dimer formation. The dose–response analysis (Figure 5) indicates that the mutant and wild‐type photoreceptor proteins are similarly responsive to UV‐B over a fluence range where the response is not saturated. The lower fold‐induction of *HY5* transcripts in GFP‐UVR8^D96N,D107N^ can be explained by the smaller amount of photoreceptor protein in the plants used for the experiments. Evidence from over‐expression lines (Favory *et al*., 2009) indicates that an increased level of UVR8 protein gives an increased magnitude of response.

The established model of UVR8 action is that the dimer acts in UV‐B photoreception, which causes monomerisation, and then the monomer interacts with COP1 to initiate signaling and hence responses (Tilbrook *et al*., 2013; Jenkins, 2014a). This model is inadequate to explain the *in vivo* UV‐B response of GFP‐UVR8^D96N,D107N^. There is, at most, only a very low concentration of dimer present in GFP‐UVR8^D96N,D107N^ plants, whereas responsiveness to UV‐B is similarly efficient to that of wild‐type GFP‐UVR8. Clearly, the physiological response of GFP‐UVR8^D96N,D107N^ does not correlate with monomer formation through UV‐B‐induced dimer dissociation. The simplest explanation is that the monomeric protein is competent in photoreception, which initiates signaling and UV‐B responses *in vivo*. Thus, the molecular and physiological phenotype of GFP‐UVR8^D96N,D107N^ indicates that dimer formation is not essential for photoreception by UVR8. UV‐B photoreception could activate monomeric UVR8^D96N,D107N^ bound to COP1 by, for example, causing a conformational change to the protein that initiates signaling. Recent studies show that photochemical events associated with UV‐B photoreception are detectable in both dimeric and monomeric UVR8 (Mathes *et al*., 2015), indicating that monomeric UVR8 is capable of UV‐B photoreception, at least *in vitro*.

The findings with the GFP‐UVR8^D96N,D107N^ mutant raise the question of whether monomeric UVR8 could be active in photoreception in wild‐type plants to initiate responses. Recent research shows that UVR8 does not behave as a simple dimer/monomer UV‐B switch under photoperiodic conditions, but establishes a photo‐equilibrium between the dimer and monomer forms (Findlay and Jenkins, 2016). Monomeric UVR8 is present in plants even at low ambient levels of UV‐B and could potentially be active in photoreception to initiate responses. At present, dimer and monomer photoreception cannot be distinguished in plants by distinct biophysical signals or associated physiological responses, and therefore further research is needed to test the intriguing possibility that monomeric UVR8 is active in photoreception in wild‐type plants.

## Experimental Procedures

### Experiments with purified proteins

Site‐directed mutagenesis was carried out using the QuikChange Site‐directed Mutagenesis kit (Stratagene, La Jolla, CA, USA) following the manufacturer's instructions and verified by sequencing. The primers used for site‐directed mutagenesis are listed in Table S2. Proteins were expressed in *E. coli* and purified as described previously (Christie *et al*., 2012). UV‐B exposure of proteins was undertaken with a narrowband UV‐B source with maximal emission at 311 nm (Philips TL20W/01RS; spectrum shown in Cloix *et al*., 2012).

Purified proteins were exposed to 1.5 μmol m^−2^ sec^−1^ narrowband UV‐B on ice for 1 h. Protein samples were prepared for electrophoresis without boiling as described (Christie *et al*., 2012) and loaded on a 7.5% SDS–PAGE gel. Gels were stained with Coomassie Blue.

Analytical SEC was performed on a Superdex 200 HR10/30 column (GE Healthcare, Little Chalfont, UK) equilibrated with wash buffer containing 50 mm Tris pH 7.5, 150 mm NaCl (or 500 mm for high‐salt samples), 1 mm β‐mercaptoethanol (β‐ME) and 0.02% sodium azide, and run at a flow rate of 0.5 ml min^−1^ at 4°C on an AKTA FPLC system (Christie *et al*., 2012). Aldolase, albumin and ovalbumin were used as standards.

### Plant material

Seeds of wild‐type *Arabidopsis thaliana* ecotype Landsberg *erecta* (L*er*) were obtained from the Nottingham Arabidopsis Stock Center. Seeds of the *uvr8‐1* mutant allele (L*er* background; Kliebenstein *et al*., 2002) were obtained from Dr Dan Kliebenstein (University of California, Davis). The *uvr8‐1/UVR8*_*pro*_ *:GFP‐UVR8* transgenic line 6‐2 was described by Kaiserli and Jenkins (2007).

Mutant UVR8 proteins were expressed in *uvr8‐1* using Agrobacterium‐mediated transformation. Mutant *UVR8* sequences were sub‐cloned into the pEZR(K)L‐C vector downstream of eGFP and the CaMV *35S* promoter (Brown *et al*., 2005; Cloix *et al*., 2012). DNA sequencing confirmed that the fusions were made correctly. At least three independent transgenic lines were selected for each fusion with a level of transgene expression comparable to that of the control GFP‐UVR8 fusion (Figure S4).

### Arabidopsis experiments

Except where indicated below, plants were grown on agar plates containing half‐strength Murashige and Skoog (MS) salts under 100 μmol m^−2^ sec^−1^ constant white light (warm white fluorescent tubes Osram) at 21°C for 7–10 days, and then either placed in darkness for 16 h or, for RT‐PCR experiments, transferred to 20 μmol m^−2^ sec^−1^ constant white light for 4 days.

Plant protein extracts were made and exposed to UV‐B using the above narrowband source as described in Heilm and Jenkins (2013). UVR8 dimer/monomer status was examined by SDS–PAGE with non‐boiled samples (Heilmann and Jenkins, 2013). Immunoblots were incubated with an anti‐GFP antibody (Clontech, Saint‐Germain‐en‐Laye, France) or anti‐UVR8 antibody (Kaiserli and Jenkins, 2007), as indicated in the figure legends.

For cross‐linking of proteins, proteins were extracted in PBS containing protease inhibitor cocktail tablets (complete, Roche, Welwyn, UK). Samples were then centrifuged for 10 min at 16 000 ***g*** at 4°C and the supernatant transferred to a fresh tube. DSP (4 mm final concentration; Thermo Scientific, Waltham, MA, USA) was then added to the extract and incubated on ice for 30 min. Immediately afterwards, protein sample buffer without reducing agent (β‐ME) or with β‐ME (5% final concentration to reverse cross‐linking) was added, and samples were boiled for 10 min before separation on a 10% SDS–PAGE and subsequent immunodetection using anti‐UVR8 antibody.

Interaction of wild‐type and mutant GFP‐UVR8 fusions with COP1 was examined by co‐immunoprecipitation (Cloix *et al*., 2012). Plants were grown on agar plates as described above and put in darkness for 16 h. The plants were treated for 3 h with 3 μmol m^−2^ sec^−1^ narrowband UV‐B. Whole cell extracts were prepared as described in Kaiserli and Jenkins (2007) in the absence or presence of 3 μmol m^−2^ sec^−1^ narrowband UV‐B. The co‐immunoprecipitation assays were carried out in the same light conditions using anti‐GFP microbeads (μMacs, 130‐091‐370, Miltenyi Biotec) as described previously (Cloix *et al*., 2012). The ‘input’ samples applied to the microbead columns and the immunoprecipitate eluates were analysed by SDS–PAGE followed by Western blotting and immunodetection using anti‐GFP (Clontech) and anti‐COP1 (kindly provided by Dr Nam‐Hai Chua; Jang *et al*., 2010) antibodies.

The RT‐PCR assays of *HY5* and *CHS* transcript levels in plant RNA samples (Figure 4b) were measured as described previously (Cloix *et al*., 2012), with the primers stated in Brown and Jenkins (2008). Plants grown as above were exposed, or not in controls, to 3 μmol m^−2^ sec^−1^ broadband UV‐B (Q‐panel UV‐B‐313 fluorescent tubes covered with cellulose acetate; spectrum shown in Cloix *et al*., 2012) for 4 h. Transcript levels of *ACTIN2* were assayed in the same cDNA samples as a control. For each gene, PCR was monitored over a range of cycle numbers to select optimal conditions for visualisation of the PCR product and quantification. Transcript levels in different RNA samples were compared using cycle numbers within the linear range of amplification.

For the dose–response experiments (Figure 5), plants were grown on agar plates as above in a 16 h light (60 μmol m^−2^ sec^−1^)/8 h dark cycle for 14 days, and transferred to darkness for 16 h prior to exposure to narrowband UV‐B at different doses as described by Brown *et al*. (2009): 20 and 40 min treatments with 0.3, 0.6 and 0.9 μmol m^−2^ sec^−1^ followed by transfer to darkness so that all tissue was harvested 2 h after the start of illumination. qRT‐PCR assays of *HY5* transcripts were undertaken similarly to Brown *et al*. (2009), but using different primers (*HY5*: forward GGCTGAAGAGGTTGTTGAGGAAC; reverse AGCATCTGGTTCTCGTTCTGAAGA. *ACTIN2*: forward GTATTGTGCTGGATTCTGGTG; reverse GAGGTAATCAGTAAGGTCACG).

For measurements of hypocotyl length, seedlings were grown for 4 days on agar plates containing half‐strength MS salts in 1.5 μmol m^−2^ sec^−1^ white light supplemented, or not in controls, with 1.5 μmol m^−2^ sec^−1^ narrowband UV‐B (Cloix *et al*., 2012). For the analysis of CHS protein, seedlings were grown under the same conditions for 7 days. Whole cell extracts were made and protein samples boiled prior to electrophoresis on a 7.5% SDS–PAGE gel. Immunoblots were probed with an anti‐CHS (Santa Cruz Biotechnology, Heidelberg, Germany) antibody. Immunoblots were stained with Ponceau S to reveal the Rubisco large subunit, which was used as a loading control.

Similar results for dimer/monomer status, COP1 interaction, hypocotyl growth suppression and gene expression were obtained for several lines expressing a particular fusion. Unless indicated otherwise, the transgenic lines used in the experiments shown are as follows: GFP–UVR8 6‐2; GFP–UVR8^R286A^ 6‐8; GFP–UVR8^R286K^ 2‐3; GFP–UVR8^D96N/D107N^ 74‐2 or 5‐2 (for the dose–response experiments); GFP–UVR8^R234A^ 16‐5; GFP–UVR8^R338A^ 9‐3 and GFP–UVR8^R146A^ line 9. The data presented are representative of at least three independent experiments.

### Transient expression in *Nicotiana benthamiana* for SEC

A single colony from Agrobacterium cells freshly transformed with the desired plasmid DNA was inoculated in 10 ml of LB medium with appropriate antibiotics and grown overnight at 28°C under constant shaking (200 rpm). When cultures had reached an OD600 of about 0.6–1.0, cells were pelleted by centrifugation at 2000 ***g*** for 10 min. The cells were then resuspended in 10 mm MgCl_2_, 10 mm MES pH 6.5 and 200 μm acetosyringone at an OD600 of 0.2 and incubated at room temperature for 3 h. The Agrobacterium medium was infiltrated into the lower side of *N. benthamiana* leaves using a syringe. The infiltrated plants were moved back into the growth room at 28°C and left for 2–3 days before examining gene expression by confocal microscopy and preparation of protein extracts.

For protein extraction, *N. benthamiana* leaf segments were frozen in liquid nitrogen and ground with a mortar and pestle. A spatula of polyvinyl‐pyrrolidone, an effective absorbent for phenolic compounds, was added as soon as the liquid nitrogen had evaporated. Once ground, the plant material was transferred to a microcentrifuge tube and approximately one volume of extraction buffer (1 mm EDTA, 10% glycerol, 5 mm DTT, 0.1% v/v Triton, 25 mm Tris‐HCl, pH 7.5) was added and vortexed to mix. Samples were centrifuged at 16 000 ***g*** for 15 min at 4°C and the supernatant was transferred to a fresh tube. The transiently expressed GFP fusions were immunoprecipitated using anti‐GFP microbeads (μMacs, 130‐091‐370, Miltenyi Biotec) as described previously (Cloix *et al*., 2012). The immunoprecipitated proteins were examined by SEC, using the same method as for the purified proteins, followed by standard SDS–PAGE and immunodetection using an anti‐GFP antibody (Clontech).

### BiFC experiments

The GFP–UVR8^D96N/D107N^ fusion was cloned into the pSPYNE and pSPYCE vectors (Walter *et al*., 2004) containing the N‐ and C‐terminal regions of YFP, respectively. Agrobacteria containing the plasmids were grown overnight as above, and resuspended together in 10 mm MgCl_2_, 10 mm MES pH 6.5 and 200 μm acetosyringone at an equivalent OD600 of 0.1 for each culture. The Agrobacterium suspension was incubated at room temperature for 2 h before infiltration into *N. benthamiana* leaves as described above. Plants were exposed to low fluence rate (1 μmol m^−2^ sec^−1^) narrowband UV‐B in a growth chamber at 21°C for approximately 60 h; controls were kept under a UV‐B cutoff filter. Leaves were examined for YFP fluorescence in at least three fields of view using a Zeiss LSM confocal microscope (Jena, Germany).

## Supporting information


**Figure S1.** Dimer/monomer status of UVR8^R286K^ examined by SEC.
**Figure S2.** Dimer/monomer status of UVR8^R146A^ and UVR8^R234A^ examined by SEC.
**Figure S3.** Dimer/monomer status of UVR8^R338A^ examined by SEC.
**Figure S4.** Expression levels of GFP‐UVR8 mutants in transgenic lines.
**Figure S5.** Dimer/monomer status of purified mutant proteins examined by SDS–PAGE with non‐boiled samples.

**Table S1.** Summary of phenotypes of UVR8 salt‐bridge amino acid mutants.

**Table S2.** Primers used for site‐directed mutagenesis.
