# Supplementary material for: Dimer/monomer status and in vivo function of salt‐bridge mutants of the plant UV‐B photoreceptor UVR8
Source: Plant J. 2016 Sep 9;88(1):71–81. doi: 10.1111/tpj.13260 (PMC5091643; doi:10.1111/tpj.13260)
Supplement: Supplementary file 1 — Figure S1. Dimer/monomer status of UVR8R286K examined by SEC. Figure S2. Dimer/monomer status of UVR8R146A and UVR8R234A examined by SEC. Figure S3. Dimer/monomer status of UVR8R338A examined by SEC. Figure S4. Expression levels of GFP‐UVR8 mutants in transgenic lines. Figure S5. Dimer/monomer status of purified mutant proteins examined by SDS–PAGE with non‐boiled samples. [file TPJ-88-71-s001.pdf]

SUPPORTING INFORMATION

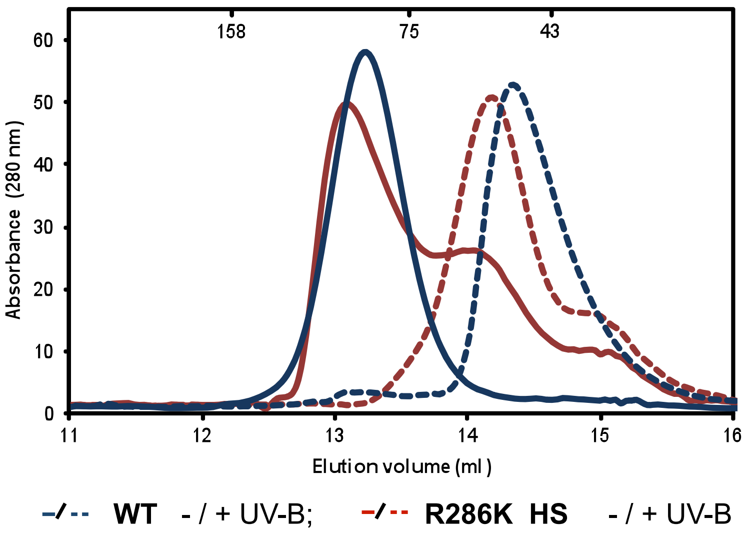

Figure S1. Dimer/monomer status of $UVR8^{R286K}$ examined by SEC.
SEC in buffer containing 500 mM NaCl of wild-type UVR8 and the $UVR8^{R286K}$ mutant exposed, or not, to 1.5 μmol m^{-2} s^{-1} narrowband UV-B for 1 h.

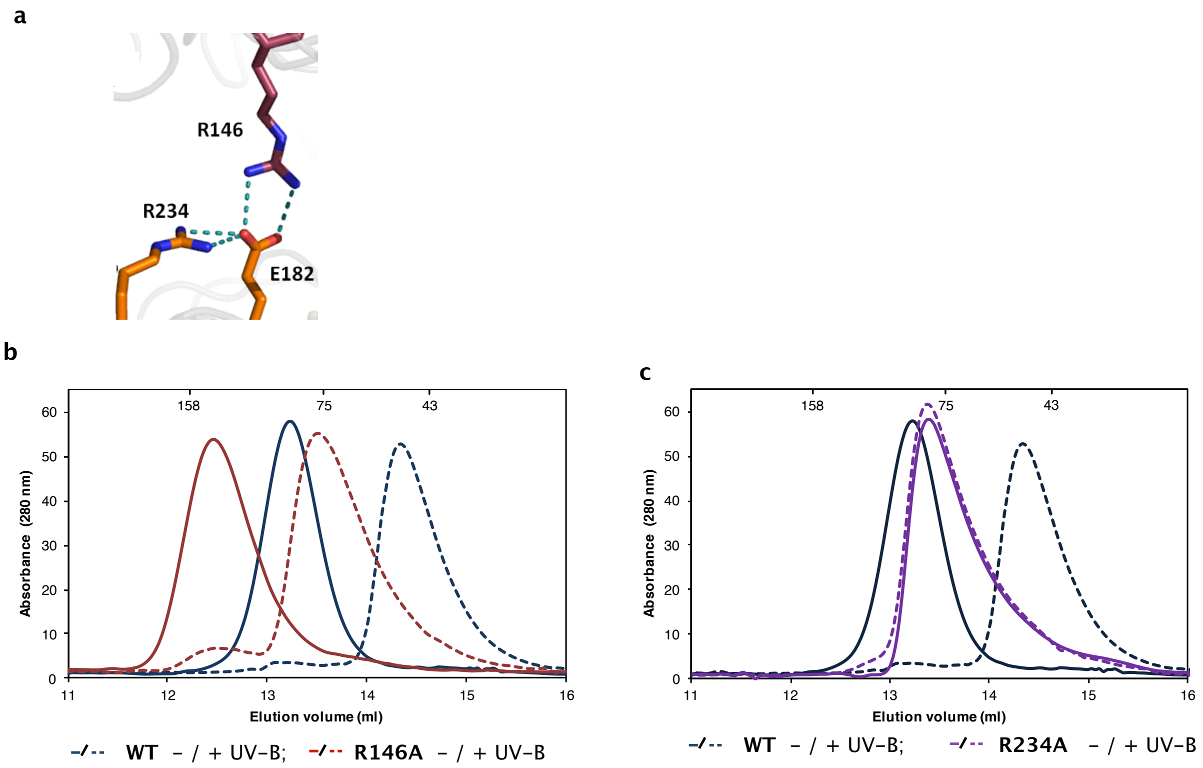

Figure S2. Dimer/monomer status of UVR8^{R146A} and UVR8^{R234A} examined by SEC.

(a) PyMol image showing inter-monomer salt bridges formed between R146 and E182 and intra-monomer salt bridges between R234 and E182.

(b) SEC of wild-type UVR8 and the UVR8^{R146A} mutant exposed, or not, to 1.5 μmol m^{-2} s^{-1} narrowband UV-B for 1 h.

(c) SEC of wild-type UVR8 and the UVR8^{R234A} mutant exposed, or not, to 1.5 μmol m^{-2} s^{-1} narrowband UV-B for 1 h.

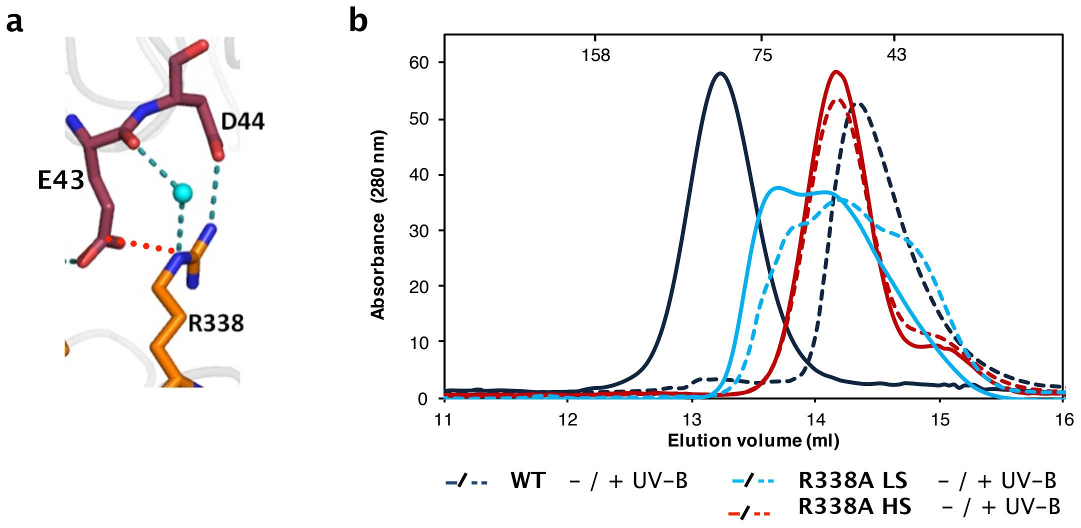

Figure S3. Dimer/monomer status of UVR8^{R338A} examined by SEC.

(a) PyMol image showing inter-monomer interactions between R338 and D44 and E43. Hydrogen bonded interactions (see text) are shown in cyan and the non hydrogen-bonded ionic interaction between R338 and E43 is shown in red.

(b) SEC of wild-type UVR8 and the UVR8^{R338A} mutant at normal NaCl concentration (LS) and of UVR8^{R338A} at high (500 mM) NaCl (HS), exposed, or not, to 1.5 μmol m^{-2} s^{-1} narrowband UV-B for 1 h.

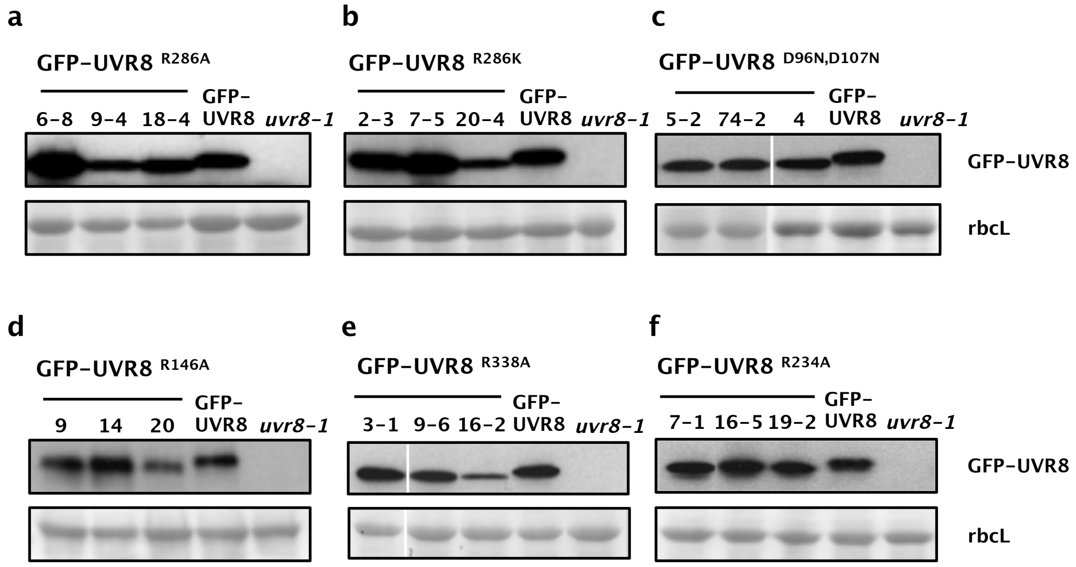

Figure S4. Expression levels of GFP-UVR8 mutants in transgenic lines. Immunoblot analysis of whole cell extracts of transgenic lines expressing the indicated GFP-UVR8 fusions. Extracts were separated by SDS-PAGE and immunoblots were probed with anti-GFP antibody. Ponceau S staining of Rubisco large subunit (rbcL) is shown as a loading control. Three independent homozygous T3 lines (numbered) were selected for each GFP-UVR8 mutant (except T2 generation for GFP-UVR8^{R146A} and one of the lines [line 4] for GFP-UVR8$^{D96N/D107N}$). The level of expression in each line was compared to that in GFP-UVR8 line 6-2, in which the level of GFP-UVR8 expression is sufficient to functionally complement *uvr8-1 (*Kaiserli and Jenkins, 2007).

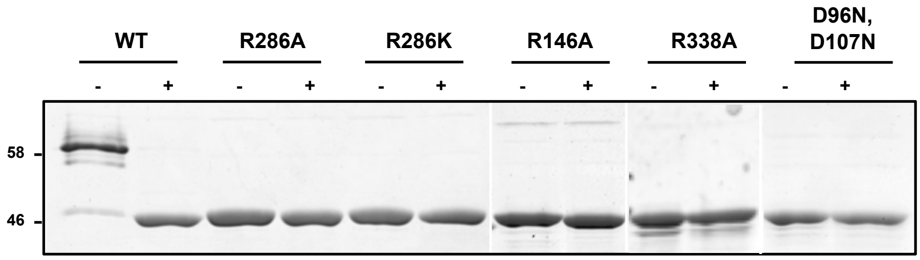

Figure S5. Dimer/monomer status of purified mutant proteins examined by SDS-PAGE with non-boiled samples.

Proteins were exposed (+) or not (-) to 1.5 μmol m^{-2} s^{-1} narrowband UV-B for 1 h. Non-boiled samples were separated by 7.5% SDS-PAGE. Proteins were stained with coomassie blue.
