# Supplementary material for: Dimer/monomer status and in vivo function of salt‐bridge mutants of the plant UV‐B photoreceptor UVR8
Source: Plant J. 2016 Sep 9;88(1):71–81. doi: 10.1111/tpj.13260 (PMC5091643; doi:10.1111/tpj.13260)
Supplement: Supplementary file 2 — Table S1. Summary of phenotypes of UVR8 salt‐bridge amino acid mutants. [file TPJ-88-71-s002.pdf]

Table S1. Summary of phenotypes of UVR8 salt-bridge amino acid mutants

Response	Wild-type	R286A	R286K	D96N,D107N	R146A	R234A	R338A
Dimer/ monomer in vitro by SEC	Dimer – UV-B; Monomer + UV-B	Constitutive monomer	Similar to wt	Constitutive monomer	As wt	Dimer; no response to UV-B	Weak dimer[d]
Dimer/ monomer in plants	Dimer – UV-B; Monomer + UV-B	Monomer/ weak dimer[b]	Weak dimer	Weak dimer - detectable only by BiFC	Weak dimer	Weak dimer/ monomer[c]	Weak dimer/ monomer[c]
UVR8-COP1 interaction in plants	UV-B induces interaction	No interaction	Constitutive interaction; increase +UV-B	Constitutive interaction increase +UV-B	Constitutive interaction; increase +UV-B	Constitutive interaction	Constitutive interaction
Functional assays in plants	UV-B induces gene expression[a] and hypocotyl growth suppression	No responses	Similar to wt	Similar to wt	Similar to wt	Very little response	Very little/no response

[a] *HY5* and *CHS* transcripts and CHS protein accumulation.
[b] Monomeric in SDS-PAGE with non-boiled sample and cross-linking assay; but weak dimer cannot be excluded.
[c] Based on SDS-PAGE with non-boiled sample.
[d] Dimer/monomer status dependent on salt concentration
