# Supplementary material for: Dimer/monomer status and in vivo function of salt‐bridge mutants of the plant UV‐B photoreceptor UVR8
Source: Plant J. 2016 Sep 9;88(1):71–81. doi: 10.1111/tpj.13260 (PMC5091643; doi:10.1111/tpj.13260)
Supplement: Supplementary file 3 — Table S2. Primers used for site‐directed mutagenesis. [file TPJ-88-71-s003.pdf]

Table S2: Primers used for site-directed mutagenesis.

Mutation		Primer Sequence
D96N	**F**	5'-ACAGTTGGGGATGGGGTAATTTTGGGAGATTAGGC-3'
	**R**	5'-GCCTAATCTCCCAAAATTACCCCATCCCCAACTGT-3'
D107N	**F**	5'-GCCATGGTAACTCAAGCAACTTGTTTACTCCGCTA-3'
	**R**	5'-TAGCGGAGTAAACAAGTTGCTTGAGTTACCATGGC-3'
R146A	**F**	5'-GTCCAGAGTTGGGGCGCCAACCAGAATGGTCA-3'
	**R**	5'-TGACCATTCTGGTTGGCGCCCCAACTCTGGAC-3'
R234A	**F**	5'-AATGGTTGCTTGTGGATGGGCGCACACAATATCAGTTTCC-3'
	**R**	5'-GGAAACTGATATTGTGTGCGCCCATCCACAAGCAACCATT-3'
R286A	**F**	5'-CCAGATTTCGGGAGGTTGGGCACATACAATGGCATTGACT-3'
	**R**	5'-AGTCAATGCCATTGTATGTGCCCAACCTCCCGAAATCTGG-3'
R286K	**F**	5'-CCAGATTTCGGGAGGTTGGAAACATACAATGGCATTG-3'
	**R**	5'-CAATGCCATTGTATGTTTCCAACCTCCCGAAATCTGG-3'
R338A	**F**	5'-CAAGTCTCATGTGGATGGGCACATACCTTGGCTGTCAC-3'
	**R**	5'-GTGACAGCCAAGGTATGTGCCCATCCACATGAGACTTG-3'
