# Supplementary material for: Dimer/monomer status and in vivo function of salt‐bridge mutants of the plant UV‐B photoreceptor UVR8
Source: Plant J. 2016 Sep 9;88(1):71–81. doi: 10.1111/tpj.13260 (PMC5091643; doi:10.1111/tpj.13260)
Supplement: Supplementary file 4 [file TPJ-88-71-s004.docx]

**Supporting Information Legends**

**Figure S1.** Dimer/monomer status of UVR8^R286K^ examined by SEC. SEC in buffer containing 500 mM NaCl of wild-type UVR8 and the UVR8^R286K^ mutant exposed, or not, to 1.5 μmol m^-2^ s^-1^ narrowband UV-B for 1 h.

**Figure S2.** Dimer/monomer status of UVR8^R146A^ and UVR8^R234A^ examined by SEC.

(a) PyMol image showing inter-monomer salt bridges formed between R146 and E182 and intra-monomer salt bridges between R234 and E182. (b) SEC of wild-type UVR8 and the UVR8^R146A^ mutant exposed, or not, to 1.5 μmol m^-2^ s^-1^ narrowband UV-B for 1 h. (c) SEC of wild-type UVR8 and the UVR8^R234A^ mutant exposed, or not, to 1.5 μmol m^-2^ s^-1^ narrowband UV-B for 1 h.

**Figure S3.** Dimer/monomer status of UVR8^R338A^ examined by SEC. (a) PyMol image showing inter-monomer interactions between R338 and D44 and E43. Hydrogen bonded interactions (see text) are shown in cyan and the non hydrogen-bonded ionic interaction between R338 and E43 is shown in red. (b) SEC of wild-type UVR8 and the UVR8^R338A^ mutant at normal NaCl concentration (LS) and of UVR8^R338A^ at high (500 mM) NaCl (HS), exposed, or not, to 1.5 μmol m^-2^ s^-1^ narrowband UV-B for 1 h.

**Figure S4.** Expression levels of GFP-UVR8 mutants in transgenic lines.

Immunoblot analysis of whole cell extracts of transgenic lines expressing the

indicated GFP-UVR8 fusions. Extracts were separated by SDS-PAGE and

immunoblots were probed with anti-GFP antibody. Ponceau S staining of

Rubisco large subunit (rbcL) is shown as a loading control. Three independent

homozygous T3 lines (numbered) were selected for each GFP-UVR8 mutant

(except T2 generation for GFP-UVR8^R146A^ and one of the lines [line 4] for

GFP-UVR8^D96N/D107N^). The level of expression in each line was compared to

that in GFP-UVR8 line 6-2, in which the level of GFP-UVR8 expression is

sufficient to functionally complement *uvr8-1* (Kaiserli and Jenkins, 2007).

**Figure S5.** Dimer/monomer status of purified mutant proteins examined by SDS-PAGE with non-boiled samples. Proteins were exposed (+) or not (-) to 1.5 μmol m^-2^ s^-1^ narrowband UV-B for 1 h. Non-boiled samples were separated by 7.5% SDS-PAGE. Proteins were stained with coomassie blue.

**Table S1.** Summary of phenotypes of UVR8 salt-bridge amino acid mutants.

**Table S2.** Primers used for site-directed mutagenesis.
